# Pain Scores Associated with Lidocaine-Tetracaine Versus Lidocaine-Prilocaine Topical Anesthesia During Facial and Cervical Microneedling: A Retrospective Single-Center Observational Analysis

**DOI:** 10.3390/medicina62061026

**Published:** 2026-05-25

**Authors:** Lidia Majewska, Karolina Dorosz, Iwona Paciepnik, Agnieszka Budzyńska

**Affiliations:** 1 ESME Clinic, Private Practice, Ul. Lwowska 1/u16, 30-548 Krakow, Poland; 2 Biological Sciences Division, University of Chicago, Chicago, IL 60637, USA; kdorosz@uchicago.edu; 3 Department of Family Medicine, Jagiellonian University Medical College, Ul. Bocheńska 4, 31-061 Krakow, Poland; iwona.paciepnik@uj.edu.pl; 4 Małopolska Burn and Plastic Surgery Center, Os. Złotej Jesieni 1, 31-826 Krakow, Poland; kursy@esmeclinic.pl

**Keywords:** topical anesthesia, lidocaine-tetracaine, lidocaine-prilocaine, microneedling, pain management, Visual Analog Scale, retrospective study

## Abstract

*Background:* Effective topical anesthesia is essential to patient comfort and adherence during minimally invasive esthetic procedures. We retrospectively reviewed pain scores recorded after microneedling in a single private clinic where two topical anesthetic formulations—lidocaine 7%/tetracaine 7% (Pliaglis) and lidocaine 2.5%/prilocaine 2.5% (Anesderm)—were used as part of standard clinical practice on different anatomical sites and under different application protocols. *Methods:* Records were reviewed from 26 healthy female patients (mean age 42 ± 4 years; range 34–48) who underwent microneedling on the face and neck during 2024 in a single private clinic. According to the established clinic protocol, which was not modified for research purposes, Pliaglis was applied to the face without additional occlusion (self-occlusive peel-off film, in accordance with the manufacturer’s recommendation) and Anesderm was applied to the neck under plastic-film occlusion (also in accordance with the manufacturer’s recommendation), both for 45 min prior to microneedling at a fixed depth of 1.25 mm. Treatment allocation was determined by clinic workflow; patients and the operator were not blinded, and the order of the two products within each session was not randomized. Post-procedural pain was recorded using a Visual Analog Scale (VAS, 0–10), with one decimal precision, separately for each anatomical site. Within-patient differences were analyzed using a paired-sample t-test, with a Wilcoxon signed-rank test as a non-parametric sensitivity analysis. *Results:* Pain scores were lower at the facial site (Pliaglis, no occlusion) than at the cervical site (Anesderm, occlusion): mean VAS 3.00 ± 0.63 vs. 5.38 ± 0.75; mean within-patient difference 2.38 points, 95% CI 1.97–2.80; paired t(25) = 11.87, *p* < 0.0001; Cohen’s d = 2.33. The Wilcoxon signed-rank test produced a concordant result (*p* < 0.0001). A within-patient pain reduction of at least 30% on the facial site relative to the cervical site was observed in 81% of patients (21/26). Both products were well tolerated, with only mild transient erythema reported. *Conclusions:* In this retrospective, non-randomized, non-blinded single-center analysis, lower pain scores were observed at the facial site (treated with lidocaine-tetracaine 7%/7% without additional occlusion, per manufacturer instructions) than at the cervical site (treated with lidocaine-prilocaine 2.5%/2.5% under occlusion, per manufacturer instructions) within the same patients. Because formulation, active-drug concentration, anatomical site, and the manufacturer-mandated occlusion technique co-varied between the two conditions, the observed difference cannot be attributed to formulation alone. These findings should be regarded as hypothesis-generating and require confirmation in prospective, randomized, split-region or split-face studies that disentangle formulation effects from site- and protocol-related factors.

## 1. Introduction

Pain management is central to minimally invasive esthetic dermatology and influences both patient satisfaction and willingness to undergo or repeat procedures [1,2]. Topical anesthetics provide effective pain relief without injections and are routinely used before procedures such as microneedling, laser treatments, and injection-based esthetic interventions.

Among contemporary topical anesthetics available for dermatologic use, eutectic mixtures of lidocaine and prilocaine (LP) and self-occlusive films of lidocaine and tetracaine (LT) are among those most widely employed in routine practice [3,4,5]. LP cream (e.g., Anesderm, EMLA) typically contains lidocaine 2.5% and prilocaine 2.5% and requires plastic-film occlusion, in accordance with the manufacturer’s instructions, to enhance transdermal penetration. LT cream (e.g., Pliaglis) contains lidocaine 7% and tetracaine 7% and is supplied as a self-occlusive formulation that dries to form a flexible peel-off membrane on the skin; per the manufacturer’s instructions, it does not require additional occlusion [6]. The two products therefore differ not only in their active-drug components and concentrations but also in the manufacturer-mandated mode of application.

Each product has been independently associated with effective topical analgesia for dermatologic procedures in earlier studies [7,8,9,10,11,12,13], and a favorable systemic safety profile has been reported for LT cream applied to surfaces of up to 200 cm^2^ for up to 90 min [14]. However, few real-world studies have directly compared these two products as they are currently used in routine clinical practice. Patient acceptance and fear of pain remain documented barriers to esthetic procedures [1,2], underscoring the practical value of comparative observational data, when interpreted with appropriate methodological caveats.

In our private esthetic dermatology clinic, both products are used as part of standard pain-management protocols during microneedling. Allocation of products to anatomical regions follows the established clinic workflow, which mirrors the manufacturer-mandated mode of application: Pliaglis on the face without occlusion, Anesderm on the neck under occlusion. The aim of the present retrospective analysis was to describe the within-patient pain scores observed in routine practice when these two products are used as currently dispensed at our clinic, with explicit recognition that the resulting comparison is confounded by anatomical site, occlusion technique, and active-drug concentration. We therefore frame the study as a hypothesis-generating description of clinical observations rather than as a comparative efficacy trial.

## 2. Methods

### 2.1. Study Design and Ethical Considerations

This study is a retrospective analysis of medical records from patients who underwent microneedling procedures at a private plastic surgery outpatient clinic. The study protocol was reviewed and approved by the Bioethics Committee of the Regional Medical Chamber in Kraków (L.dz. OIL/KBL/9/2025). Data collection and analysis were conducted in accordance with the Declaration of Helsinki and Good Clinical Practice guidelines. As this was a retrospective analysis of standard clinical practice, no additional interventions were performed beyond routine care. All patients had previously provided written informed consent for the use of their anonymized data for scientific and statistical purposes as part of the clinic’s standard consent process. All data were fully anonymized prior to analysis.

### 2.2. Patient Selection

We reviewed the medical records of 26 healthy female patients aged 34–48 years (mean age 42 ± 4 years) who underwent microneedling treatment on the face and neck between January and December 2024. All patients had mild to moderate facial skin aging (Glogau scale score ≥ 2) and Fitzpatrick skin types I–III and sought skin-quality improvement through microneedling. Patients were included if they had no known hypersensitivity to lidocaine, tetracaine, prilocaine, or other amide/ester local anesthetics; no active dermatological conditions or infections in the treatment areas; and no esthetic procedures within the previous four weeks in the treated regions. Exclusion criteria were contraindications to topical anesthetics, active skin infections, and pregnancy or breastfeeding. All participants were in good general health without significant comorbidities. Indications for microneedling included improvement of skin texture, reduction in fine lines, and overall skin rejuvenation. Prior to treatment, all patients provided written informed consent for the procedure and for the use of their anonymized data for scientific and statistical purposes.

### 2.3. Anesthetic Application Protocol

All patients received topical anesthesia according to the clinic’s standard protocol, which was not modified for research purposes, and which mirrors the manufacturer-mandated mode of application of each product. Anesderm (lidocaine 2.5%/prilocaine 2.5% cream) was applied to the neck region under plastic-film occlusion, as required by the manufacturer to enhance penetration through the stratum corneum. Pliaglis (lidocaine 7%/tetracaine 7% cream) was applied to the face region without additional occlusion, because this self-occlusive formulation forms a flexible peel-off membrane upon exposure to air and the manufacturer’s instructions explicitly advise against additional occlusion. Both products were applied in a uniform layer per manufacturer guidelines and left in place for 45 min before careful removal with sterile gauze.

We acknowledge that this protocol allocates the two products to different anatomical sites, uses different occlusion methods, and involves products with markedly different active-drug concentrations (7%/7% vs. 2.5%/2.5%). The asymmetry in occlusion technique reflects an intrinsic feature of each product rather than a design choice specific to our clinic; nonetheless, the comparison of end-of-procedure VAS scores cannot separate the contribution of the active drugs from that of their delivery systems. The implications for interpretation are addressed in Section 2.6 and Section 5.

### 2.4. Microneedling Procedure

Following anesthetic removal, microneedling was performed using the Dermapen 4 device with a fixed needle depth of 1.25 mm across both the facial and the cervical treatment areas. The procedure was performed in a controlled clinical environment by the same physician for all patients, with three complete passes applied in systematic horizontal, vertical, and diagonal directions. Standardization of operator and device parameters across sessions controlled for variation in technique. It did not, however, control for differences in skin thickness, vascularization, or innervation density between facial and cervical skin, which may independently influence the nociceptive response to microneedling at a fixed needle depth and which contribute to the limitations described in Section 5.

### 2.5. Assessment of Anesthetic Efficacy

**Figure 5:**
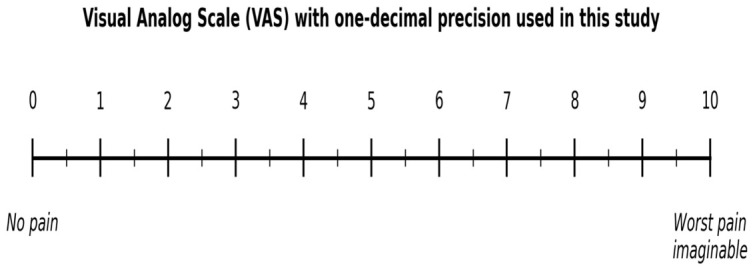
Visual Analog Scale (VAS) with one-decimal markings used in this study.

Pain was assessed immediately after each procedure, as part of routine post-procedure documentation, using a Visual Analog Scale (VAS) ranging from 0 (no pain) to 10 (worst pain) (Figure 5). Patients rated their pain levels separately for the facial and cervical regions. A modified VAS recording one decimal place was used to allow finer-grained pain assessment. Adverse effects such as erythema, burning, or irritation were recorded per the standard clinical protocol. Pain assessment was open-label: neither the patient nor the operator was blinded to the products used, and the order of pain rating between the two sites was not standardized.

### 2.6. Statistical Analysis

Descriptive statistics (mean, standard deviation, median, minimum, maximum) were computed for VAS scores at each anatomical site. Within-patient pain scores at the two sites were compared using a paired-sample *t*-test. Because the paired observations differ not only in formulation but also in anatomical site, occlusion technique, and active-drug concentration, the test cannot distinguish among these effects. The result is therefore interpreted as a within-patient difference between two non-equivalent conditions, not as an unconfounded estimate of formulation-attributable analgesia.

Because VAS data may deviate from normality in small samples, and because the assumption of equivalent measurement conditions across paired observations is not strictly met in this design, a Wilcoxon signed-rank test was performed as a non-parametric sensitivity analysis. Cohen’s d for paired samples was calculated as a measure of effect size. Two-sided *p*-values are reported, with significance set at *p* < 0.05. Visualizations (box plot, paired scatter, and histogram) were generated to illustrate the distribution of pain scores. All analyses were performed in Python (version 3.9) using NumPy and SciPy.

## 3. Results

### 3.1. Pain Scores Summary

Mean VAS pain scores were 5.38 ± 0.75 at the cervical site (Anesderm, occlusion; median 5.30, range 4.0–6.8) and 3.00 ± 0.63 at the facial site (Pliaglis, no occlusion; median 3.00, range 1.8–4.5). Individual scores for all 26 patients are summarized in Table 1.

**Figure 1:**
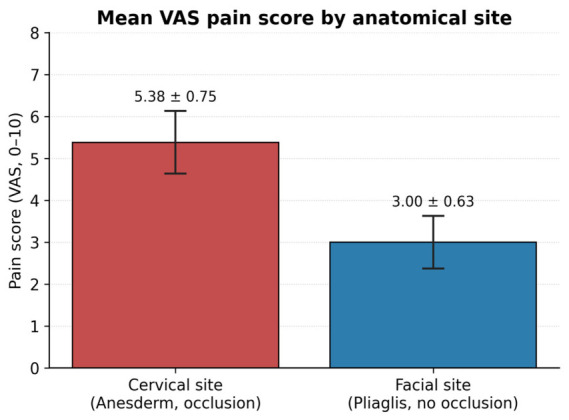
Mean VAS pain scores at the cervical site (Anesderm, occlusion) and the facial site (Pliaglis, no occlusion). Error bars represent ± 1 SD.

As illustrated in Figure 1, mean pain scores at the facial site were lower than at the cervical site, with limited overlap of standard-deviation error bars between the two conditions, indicating a consistent within-patient difference.

### 3.2. Statistical Analysis

The within-patient pain score difference between the two conditions was statistically significant. The paired *t*-test yielded t(25) = 11.87 with *p* < 0.0001 and a 95% confidence interval for the mean difference of 1.97 to 2.80 VAS points; the corresponding paired Cohen’s d was 2.33, indicating a very large effect size for the bundled comparison. The Wilcoxon signed-rank sensitivity analysis produced a concordant result (*p* < 0.0001), supporting the robustness of the within-patient difference to potential deviations from the assumptions of the parametric test. As discussed in Section 2.6 and Section 5, however, neither test can attribute the difference to formulation alone.

### 3.3. Distribution of Pain Scores

**Figure 2:**
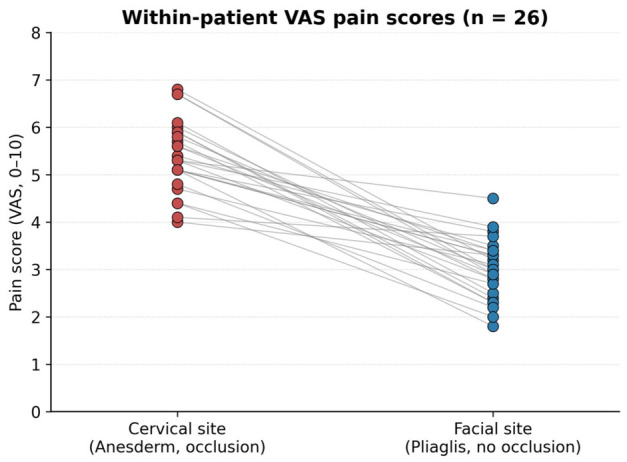
Within-patient paired VAS pain scores at the cervical site (Anesderm, occlusion) and the facial site (Pliaglis, no occlusion). Each line connects the two scores recorded for a single patient.

Figure 2 presents within-patient paired pain scores. Pain scores at the facial site clustered in the 1.8–4.5 range, while scores at the cervical site clustered in the 4.0–6.8 range, with no overlap between conditions in 25 of 26 patients. The pattern of lower facial-site scores was observed across all 26 patients.

### 3.4. Box Plot Analysis

**Figure 3:**
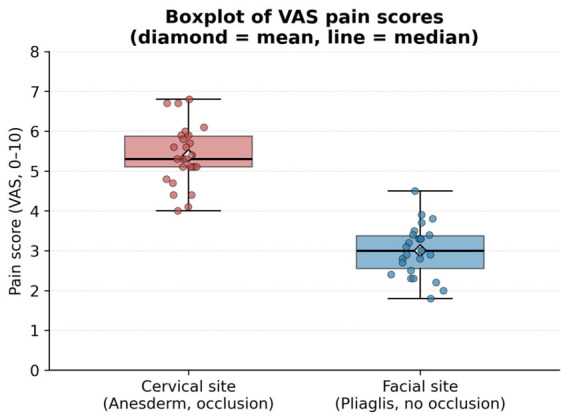
Box plot of VAS pain score distributions for the cervical and facial sites. The diamond marker indicates the mean and the horizontal line indicates the median; individual scores are jittered horizontally for visibility.

Figure 3 presents a box plot comparison of the two distributions. The interquartile range at the facial site (Pliaglis, no occlusion) was narrower and positioned lower on the VAS than at the cervical site (Anesderm, occlusion), indicating both lower median pain scores and reduced within-condition variability. The median VAS at the facial site was 3.00 and at the cervical site was 5.30, corresponding to a 43% reduction in median pain score across these two non-equivalent conditions.

### 3.5. Frequency Distribution

**Figure 4:**
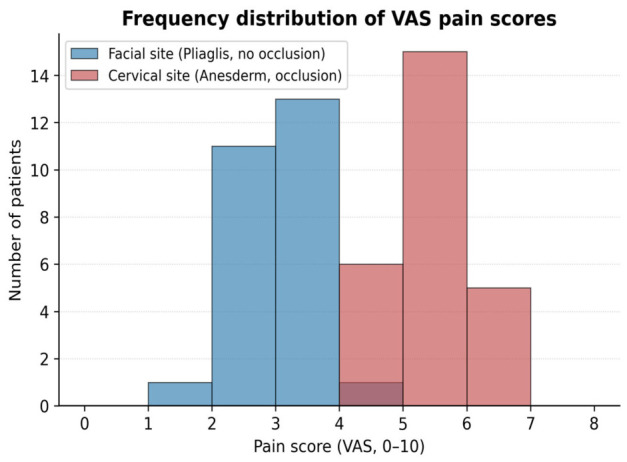
Histogram of VAS pain score frequencies at the facial and cervical sites.

The histogram in Figure 4 illustrates the frequency distribution of pain scores in one-point intervals. Most patients reported facial-site (Pliaglis) scores in the 2–4 range. Cervical-site (Anesderm) scores predominantly fell in the 5–6 range, with additional groups in the 4–5 and 6–7 ranges. No facial-site score exceeded five, and no cervical-site score fell below four.

### 3.6. Within-Patient Pain Reduction

In 21 of 26 patients (81%), the within-patient pain score recorded at the facial site (Pliaglis, no occlusion) was at least 30% lower than the score recorded at the cervical site (Anesderm, occlusion). Because the two sites also differed in baseline nociceptive sensitivity, occlusion technique, and active-drug concentration, this proportion describes a combined within-patient response to two non-equivalent conditions and should not be interpreted as a formulation-specific responder rate. The mean within-patient difference of 2.38 VAS points (95% CI 1.97–2.80) exceeds the minimum clinically important difference of approximately 1.3 points reported in the pain literature for the VAS (Figure 5).

### 3.7. Adverse Effects

No severe adverse reactions were reported for either product. Mild transient erythema and brief burning sensations were noted in some patients but resolved within minutes after the procedure, consistent with the known safety profiles of these products when used according to manufacturer guidelines.

## 4. Discussion

In this retrospective single-center analysis of routine clinical practice, pain scores recorded at the facial site (treated with self-occlusive lidocaine 7%/tetracaine 7% cream without additional dressing, in accordance with manufacturer instructions) were lower than those recorded at the cervical site (treated with lidocaine 2.5%/prilocaine 2.5% cream under plastic-film occlusion, also in accordance with manufacturer instructions) within the same patients during the same microneedling session. The mean within-patient difference of 2.38 VAS points exceeded the minimum clinically important difference of approximately 1.3 points reported in the pain literature for the VAS, and the corresponding effect size was very large (Cohen’s d = 2.33). However, several features of the study design substantially constrain the interpretation of this finding.

The central methodological caveat is that the two anesthetic products were not compared under equivalent conditions. The formulations differ in the identity and concentration of their active drugs (lidocaine-tetracaine 7%/7% vs. lidocaine-prilocaine 2.5%/2.5%); they were applied to different anatomical sites with documented differences in skin thickness, vascularity, innervation density, and procedural sensitivity [15]; and they were applied using different occlusion strategies that, while both consistent with manufacturer instructions, are themselves an intrinsic feature of each product (self-occlusive peel-off film vs. plastic-film occlusion). The same fixed needle depth (1.25 mm) may also produce non-equivalent nociceptive stimulation in facial versus cervical skin. The observed pain difference therefore reflects the combined effect of formulation, concentration, anatomical site, occlusion mechanism, and procedural factors. Our data do not permit attribution of the pain difference to anesthetic formulation alone.

Within these constraints, our observations are consistent with prior controlled trials reporting effective analgesia with LT cream during dermatologic procedures, including photodynamic therapy [8], laser CO_2_ dermabrasion [9], laser-assisted tattoo removal [10], non-ablative laser treatment [11], and laser-assisted hair removal [12]. LP cream has likewise been shown to provide effective topical anesthesia under occlusion in earlier studies [3,4,5]. The pharmacokinetic profile of LT cream has been evaluated in dedicated studies, with plasma levels of lidocaine and tetracaine below the limit of quantification when applied to surfaces of up to 200 cm^2^ for up to 90 min [14], indicating a favorable systemic safety profile.

Several factors plausibly contribute to the lower facial pain scores observed in our patients. The higher active-drug concentration of LT cream (7%/7% vs. 2.5%/2.5%) may yield deeper or more rapid anesthetic effect, the peel-off film-forming property of LT may enhance drug delivery through the stratum corneum, and facial skin may have lower baseline nociceptive sensitivity than cervical skin at a fixed needle depth. Our retrospective design cannot apportion the contribution of these factors or separate formulation effects from site and protocol effects. We therefore do not interpret our results as evidence that LT is pharmacologically superior to LP.

The findings have practical implications limited to the specific clinical setting in which the data were collected: under the existing clinic protocol—which mirrors the manufacturer-mandated mode of application of each product—patients consistently report lower pain on the face than on the neck. They do not support broader extrapolation to other dermatologic or esthetic procedures, including laser treatments, dermal filler injections, botulinum toxin injections, platelet-rich plasma, chemical peels, dermaplaning, or radiofrequency microneedling. These procedures involve different nociceptive mechanisms, were not evaluated in the present study, and have been compared in dedicated published trials [7,10,12,16,17]; selection of topical anesthetic for such procedures should be guided by those trials rather than extrapolated from the present results.

Patient acceptance and fear of pain are well-recognized barriers to esthetic procedures [1,2], which underscores the value of robust comparative data on topical anesthetic formulations. The present results may help motivate, but cannot substitute for, prospective comparative studies designed to address the methodological gaps identified above.

## 5. Limitations and Future Directions

This study has several important limitations that should temper the interpretation of its findings.

Bundled comparison. The comparison between LT and LP is confounded by non-equivalent anatomical and procedural conditions. The two products were applied to different anatomical sites (face vs. neck) with different occlusion strategies (self-occlusive peel-off vs. plastic-film occlusion) and contain markedly different active-drug concentrations (7%/7% vs. 2.5%/2.5%). Although both products were applied in accordance with their respective manufacturer instructions, the asymmetry in occlusion technique is an intrinsic feature of each product rather than a separable design factor; the comparison therefore cannot isolate the contribution of the active drugs from the contribution of their delivery systems. Microneedling was performed at a fixed depth of 1.25 mm at both sites despite known differences in skin thickness, innervation, and vascularity between facial and cervical skin. As a result, the observed pain difference cannot be attributed to anesthetic formulation alone; formulation, concentration, anatomical site, occlusion technique, and procedural factors are jointly implicated, and our design cannot disentangle them.

Retrospective, non-randomized, non-blinded design. This was a retrospective review of standard clinical care. Neither the operator nor the patients were blinded to the products used, and treatment order within each session was determined by clinic workflow rather than randomized. Patient expectation, anatomical context, and order effects may all have influenced reported VAS scores.

Limited statistical power and external validity. The sample comprised 26 women treated by a single physician at a single private clinic with Fitzpatrick skin types I–III. The findings may not generalize to other settings, operators, sex distributions, or skin phototypes.

Statistical assumptions. Although the paired t-test is appropriate for within-patient comparisons in principle, its assumption that paired measurements differ only in the manipulated factor is not strictly met here, because each pair differs in formulation, anatomical site, occlusion, and concentration concurrently. We therefore present the analysis as a description of within-patient pain difference between two non-equivalent conditions rather than as a formulation-specific effect estimate. The Wilcoxon signed-rank sensitivity analysis indicated that the within-patient difference is robust to deviations from normality but does not address the more fundamental confounding of formulation with site and protocol.

Subjective and time-limited assessment. Pain assessment using a single immediate post-procedure VAS score, while widely accepted, is subjective and does not capture onset time, duration of effect, or the trajectory of pain over the course of the procedure.

Future research should address these limitations through prospective, randomized, blinded designs. A split-face or split-region protocol applying both products to the same anatomical site (with randomized side allocation and blinded pain assessment) would directly address the formulation question. A randomized parallel-group study at a single anatomical site would provide a complementary test, although the manufacturer-mandated occlusion asymmetry between products would still need to be accounted for. Quantitative sensory testing or other objective neurophysiological measures could supplement subjective VAS reporting. Economic analyses comparing cost-effectiveness of different anesthetic protocols would also be informative.

## 6. Conclusions

This retrospective single-center analysis observed lower VAS pain scores at the facial site treated with lidocaine-tetracaine 7%/7% (without additional occlusion, per manufacturer instructions) than at the cervical site treated with lidocaine-prilocaine 2.5%/2.5% (under occlusion, per manufacturer instructions) during microneedling sessions performed in the same patients on the same day. Because the two conditions also differed in anatomical site, active-drug concentration, and the manufacturer-mandated occlusion technique, the observed difference cannot be attributed to formulation alone. The findings are best interpreted as a within-patient association between two non-equivalent routine clinical conditions, not as evidence of pharmacologic superiority of one formulation over the other. Confirmation requires prospective, randomized, blinded comparative trials in which formulation is varied independently of anatomical site and application protocol. Clinicians selecting topical anesthetic protocols for microneedling and other esthetic procedures should rely on dedicated controlled studies and weigh procedure type, anatomical region, occlusion requirements, and patient-specific factors when interpreting any observational comparison, including this one.

## Figures and Tables

**Table 1:** Within-patient VAS pain scores at the cervical site (Anesderm, occlusion) and the facial site (Pliaglis, no occlusion).

Patient	VAS—Anesderm (Neck, Occlusion)	VAS—Pliaglis (Face, No Occlusion)
1	5.9	2.4
2	5.4	3.5
3	6.0	2.8
4	6.7	3.0
5	5.3	2.8
6	5.3	4.5
7	6.8	3.2
8	6.1	2.5
9	5.1	3.8
10	5.9	2.3
11	5.1	3.3
12	5.1	1.8
13	5.7	2.3
14	4.0	3.3
15	4.1	3.7
16	5.1	3.3
17	4.7	3.1
18	5.8	3.0
19	4.8	2.2
20	4.4	2.7
21	6.7	2.9
22	5.3	3.9
23	5.6	3.4
24	4.4	2.0
25	5.1	3.4
26	5.6	2.9

## Data Availability

The datasets used and analyzed during the current study are available from the corresponding author on reasonable request.
